# The prolyl isomerase Pin1 stabilizes NeuroD during differentiation of mechanoreceptors

**DOI:** 10.3389/fcell.2023.1225128

**Published:** 2023-09-18

**Authors:** Liqun Zhao, Steven H. Fong, Qiaoyun Yang, Yun-Jin Jiang, Vladimir Korzh, Yih-Cherng Liou

**Affiliations:** ^1^ Department of Biological Sciences, Faculty of Science, National University of Singapore, Singapore, Singapore; ^2^ Genes and Development Division, Institute of Molecular and Cell Biology, Agency for Science, Technology and Research (A-STAR), Singapore, Singapore; ^3^ Institute of Molecular and Genomic Medicine, National Health Research Institutes, Zhunan, Taiwan

**Keywords:** prolyl isomerase Pin1, NeuroD, Zebrafish, cis-trans isomerization, mechanoreceptors

## Abstract

The peptidyl prolyl *cis-trans* isomerase Pin1 plays vital roles in diverse cellular processes and pathological conditions. NeuroD is a differentiation and survival factor for a subset of neurons and pancreatic endocrine cells. Although multiple phosphorylation events are known to be crucial for NeuroD function, their mechanisms remain elusive. In this study, we demonstrate that zebrafish embryos deficient in Pin1 displayed phenotypes resembling those associated with NeuroD depletion, characterized by defects in formation of mechanosensory hair cells. Furthermore, zebrafish Pin1 interacts with NeuroD in a phosphorylation-dependent manner. In Pin1-deficient cell lines, NeuroD is rapidly degraded. However, the protein stability of NeuroD is restored upon overexpression of Pin1. These findings suggest that Pin1 functionally regulates NeuroD protein levels by post-phosphorylation *cis-trans* isomerization during neuronal specification.

## Introduction

Pin1, a peptidyl-prolyl *cis-trans* isomerase (PPIase), catalyzes the intrinsically slow *cis-trans* isomerization of phosphorylated serine or threonine preceding proline (pSer/Thr-Pro) motifs in a subset of proteins (Fischer, 1994; Yaffe et al., 1997; Hunter, 1998). Accumulating evidence supports the significant role of Pin1 in post-phosphorylation regulation of various cellular processes, including cell cycle progression, gene transcription, neuronal differentiation, regulation of substrate stability, immune response to microbial infection, and also pathological conditions, such as cancers and neuronal diseases (Crenshaw et al., 1998; Zhou et al., 1999; Liou et al., 2003; Eckert et al., 2005; Yeh and Means, 2007; Siepe and Jentsch, 2009; Ghosh et al., 2013).

The *cis-trans* conformational change regulated by Pin1 influences diverse biological functions, as evidenced by studies in several model organisms. In vertebrates, Pin1 knockout mice exhibit cyclin D1-null phenotypes and progressive age-dependent neuropathy, highlighting a pivotal role of Pin1 in cell proliferation and protecting against age-dependent neurodegeneration (Liou et al., 2002; Liou et al., 2003). Interestingly, Ibarra et al. showed embryos injected with EGFP-Pin1 mRNA induced p53-dependent apoptosis at prim-5 stage (24 hpf) brain of zebrafish (Ibarra et al., 2017). Furthermore, studies on the novel Pin1-like parvulins isolated from *Trypanosoma brucei* further supports the role of Pin1 in cell growth regulation (Goh et al., 2010); a further study also suggested that Pin1-mediated signaling mechanism plays a different role in protozoan parasites (Erben et al., 2012). In plants, *Arabidopsis* Pin1 (Pin1At) regulates phosphorylation-dependent prolyl *cis-trans* isomerization of key transcription factors in flowering regulatory mechanism (Wang et al., 2010). Furthermore, Pin1At catalyzes the conformational dynamics of phosphorylated PIN1 and affects PID- and PP2A-mediated regulation of PIN1 polar localization, which correlates with the regulation of root gravitropism (Xi et al., 2016). Although loss of Pin1 in mice displays several age-dependent neurodegeneration phenotypes, its function in neuronal specification during vertebrate development has yet to be characterized. By using zebrafish as a model organism, we hope to shed some light on the function of Pin1 in neurogenesis during vertebrate development.

Proneural genes encoding transcription factors of the basic helix-loop-helix (bHLH) class are sequentially expressed during neurogenesis in vertebrates (Villares and Cabrera, 1987; Murre et al., 1989). NeuroD (Nrd), a bHLH neuron differentiation factor induces neuronal differentiation, regulates the development of endocrine cells and is strongly expressed in the pancreatic cells (Lee et al., 1995; Blader et al., 1997; Lee, 1997; Naya et al., 1997; Korzh et al., 1998). In mice, Nrd is required for the formation of granule cells in the hippocampus and cerebellum of the CNS and is essential for the development of sensory neurons in the inner ear of mice (Miyata et al., 1999; Kim et al., 2001). In zebrafish, *nrd* is expressed in the lateral line neuromasts and is essential for differentiation of the hair cells of the posterior lateral line (PLL) of zebrafish embryos (Sarrazin et al., 2006). The neurogenic activity of Nrd is regulated by phosphorylation of its multiple Ser/Pro motifs by ERK2 or GSK3β (Marcus et al., 1998; Moore et al., 2002; Khoo et al., 2003).

The zebrafish lateral line (LL) is a mechanosensory organ involved in the detection of displacement waves in the water, which allows for schooling behaviors and predator/prey detection. The LL system arises from ectodermal placodes and develops anterior and posterior to the optic placode generating the anterior and posterior lateral line systems (ALL and PLL) (Metcalfe et al., 1985). The PLL placode gives rise to a stationary population which forms the PLL ganglion, and a migratory PLL primordium containing proneuromasts to be deposited in clusters (mantle and support cells) towards the tail. Some support cells differentiate into mechanosensory hair cells in a process regulated by a cascade of bHLH factors, where the master regulator protein Atoh1 acts upstream of Nrd (Metcalfe et al., 1985; López-Schier and Hudspeth, 2006; Ghysen and Dambly-Chaudière, 2007; Millimaki et al., 2007; Go et al., 2010).

In this study, we provide novel evidence that depletion of Pin1 in zebrafish embryos resulted in PLL defects resembling Nrd deficient embryos, affecting hair cell specification in neuromasts. Additionally, we further identify Nrd as a novel Pin1 substrate, and the interaction between Pin1 with Nrd *via* the pSer/Thr-Pro motifs is crucial for maintaining Nrd stability. Our findings suggest that Pin1 regulates Nrd function through post-phosphorylation *cis-trans* isomerization in neuromasts hair cell specification. Furthermore, our study adds to the genetic evidence supporting the role of Pin1 in normal neuronal function, beyond it known role in neurodegenerative diseases.

## Materials and methods

### Rapid amplification of cDNA ends (RACE)

Full-length zebrafish *pin1* (NM_200748) was cloned using the SMART™ RACE cDNA amplification kit (BD Biosciences). 5′-Gene specific primer: 5′-GGCCGC​TCC​CAC​TGA​CTC​GCA​TTG​GT-3′; 3′-Gene specific primer: 5′-GAC​CCT​CGT​CCT​GGA​GAG​AGG​AGA​AC-3′.

### Maintenance and staging of zebrafish

Zebrafish were maintained at 28°C under standard conditions according to the rules of IACUC (Biopolis IACUC application #050096) and regulations of the Fish Facility of the IMCB. The embryos were staged as described (Kimmel et al., 1995).

### Semiquantitative RT-PCR

RNA extraction from embryos at the stages indicated (Figure 2A) was performed using Trizol (Invitrogen). Subsequently, cDNA was synthesized using SuperScript reverse transcriptase (Invitrogen). The cDNAs from various embryonic stages served as templates for amplifying the zebrafish *pin1* transcript. The Primers used were.

*β-actin* sense: 5′-GCA​CGA​GAG​ATC​TTC​ACT​CCC​CTT​G-3′;
*β-actin* antisense: 5′-CAT​CAC​CAG​AGT​CCA​TCA​CAA​TAC​C-3′;
*pin1* sense: 5′-ATG​TCC​GAT​GAC​GAC​GAG​AAG​CT-3′;
*pin1* antisense: 5′-GCG​TGT​GAT​GTT​CTC​CTC​TC-3′.

### Whole mount *in situ* hybridization

Whole-mount *in situ* hybridization of zebrafish embryos was performed following the protocol described in the zebrafish book (Westerfield, 2000). Antisense/sense RNA probes were produced with a dioxygenin RNA labeling kit (Roche). For 48 hpf embryos, the hybridization temperature was reduced from 68°C to 58°C and the staining time was extended to 3–4 h. These modified conditions allowed specific detection of the zebrafish Pin1 transcript on the lateral line neuromasts.

### Microinjection

Morpholino antisense oligonucleotides (Gene Tools Plc, United States) were injected at different concentrations ranging from 0.4 to 0.8 pmol in 1 × Danieau buffer (58 mM NaCl; 0.7 mM KCl; 0.4 mM MgSO4; 0.6 mM Ca(NO_3_)_2_; 5.0 mM Hepes pH 7.6). Needles for microinjection were prepared using the Sutter Micropipette puller P-97 (Sutter Instruments Co, United States). Microinjection was performed on 1-2 cell stage embryos using Picoinjector PLI-100 (Medical Systems Corp, Greenvale, NY, United States). MO sequences: *pin1* MO1: 5′-ACG​GCA​GCT​TCT​CGT​CAT​CGG​ACA​T-3′; *pin1* MO2: 5′-GAT​TGC​AGG​ACG​GCT​CGG​TTC​GG-3′; Standard control MO: 5′-CCT​CTT​ACC​TCA​GTT​ACA​ATT​TAT​A-3′.

### Cell culture and transfection

Cell lines used: wild-type HEK 293T and HEK 293T stably expressing control siRNA and Pin1 siRNA, wild-type MEF and Pin1^−/−^ MEF cells and SH-SY5Y cells. HEK 293T and MEF cells were maintained at 37°C and 5% CO_2_ in Dulbecco’s Modified Eagle Medium (DMEM) with 10% FBS (Hyclone), 2% 750 g/L NaHCO_3_ (Gibco), 100 U/mL penicillin G (Gibco) and 100 μg/mL streptomycin sulfate (Gibco). SH-SY5Y cells were grown under similar conditions in a 1:1 mixture of Eagle’s Minimum Essential Medium (MEM, Hyclone) with non-essential amino acids and Ham’s F12 medium (Hyclone) supplemented with 10% FBS (Hyclone). HEK 293T cells were transfected by calcium phosphate method. MEF and SH-SY5Y cells were transfected with Lipofectamine™ 2000 (Invitrogen) according to manufacturer’s protocol.

The stable Pin1 siRNA expression is a retrovirus-mediated RNA interference targeting Pin1. The following has been added into M&M. Establish stable Pin1-siRNA HEK 293T cells. The retrovirus was generated as described in (Ryo et al., 2005). In brief, PLAT-E cells were transfected with pSuper-puro-Pin 1-ShRNA or control -ShRNA and vesicular stomatitis virus G (VSVG) vectors (kindly provided by Dr. Ryo, 2005), using Lipofectamine 2000 (Invitrogen). Culture supernatants of PLAT-E cells were collected 48 h following transfection with retroviral vectors. HEK-293T cells were infected in the presence of 10 ug/mL Polybrene. The stable clones were selected by continuous growth in 2 μg/mL puromycin (Sigma #P8833).

### Immunostaining

SH-SY5Y cells were transfected with GFP-tagged zebrafish Pin1 and HA-Nrd and seeded on coverslips. After 48 h, the cells were fixed with 4% paraformaldehyde/PBS for 30 min and blocked with 3% BSA; 0.1% Triton/PBS for 30 min at room temperature. Subsequently, the cells were incubated with polyclonal anti-GFP and monoclonal anti-HA antibodies (Santa Cruz Biotechnology) for 1 h at room temperature, followed by Alexa-Flour-488 anti-rabbit and Alexa-Fluor-568 anti-mouse antibodies (Molecular probes) for 1 h at room temperature. Nuclear staining were performed using Hoechst 33,342 (Molecular probes) for 10 min at room temperature. Finally, cells were then mounted with FluorSave™ reagent (Calbiochem) and examined under confocal fluorescence microscope (Zeiss META LSM510).

### Co-immunoprecipitation assay

HA-tagged Nrd were co-transfected either with FLAG-tagged zebrafish Pin1 or FLAG-vector control. Cell lysates were collected in mammalian cell lysis buffer (50 mM Hepes pH 7.4; 10% glycerol; 1% Triton X-100; 100 mM NaCl and 0.5 mM MgCl_2_) supplemented with protease and phosphatase inhibitors. Supernatants were incubated with FLAG-M2 beads (Sigma) for 1.5 h at 4°C. Bound proteins were eluted by 2 × SDS loading dye. The samples were analyzed on SDS-PAGE and Western blotting with polyclonal anti-HA antibody (Zymed) and monoclonal anti-FLAG antibody (Santa Cruz Biotechnology).

### GST-pulldown assay

The recombinant GST or GST-tagged zebrafish Pin1 proteins were immobilized on Glutathione Sepharose 4B beads (Amersham Biosciences). The beads were then incubated with HEK 293T cell lysates overexpressing HA-tagged Nrd or Nrd mutants at 4°C for 3 h. Bound proteins were eluted by 2 × SDS loading dye and analyzed by Western blotting with polyclonal anti-HA antibody (Zymed).

### Protein stability assay

For stability assays, HA-Nrd/HA-Nrd5A were transfected into HEK 293T control siRNA and HEK 293T Pin1 siRNA cells; or MEFs WT and Pin1^(−/−)^ cells, respectively. The cells were treated with cycloheximide (100 μg/mL; Sigma) after 24 h of transfection to inhibit *de novo* protein synthesis. The cells were then harvested using mammalian cell lysis buffer supplemented with protease and phosphatase inhibitors at the different time points. Equal amounts of total proteins for each time point were loaded onto SDS-PAGE and analyzed by Western blotting against monoclonal anti-HA antibody (Santa Cruz Biotechnology) and monoclonal anti α-tubulin antibody (Sigma-Aldrich).

To investigate if re-introduction of Pin1 could stabilize Nrd in Pin1 depleted cells, HA-Nrd was co-transfected with FLAG-tagged zebrafish Pin1 or infected with adenovirus encoding human Pin1 into HEK 293T control siRNA and Pin1 siRNA cells. The cells were treated with cycloheximide and harvested as above to be analyzed by Western blotting with monoclonal anti-HA (Santa Cruz Biotechnology), monoclonal anti-FLAG (Santa Cruz Biotechnology) and monoclonal antibody α-tubulin (Sigma Aldrich) antibodies.

Densitometry was performed on scanned immunoblot images using the ImageJ gel analysis tool.

## Results

### Expression of *pin1* during zebrafish embryogenesis and adult tissues

**FIGURE 1:**
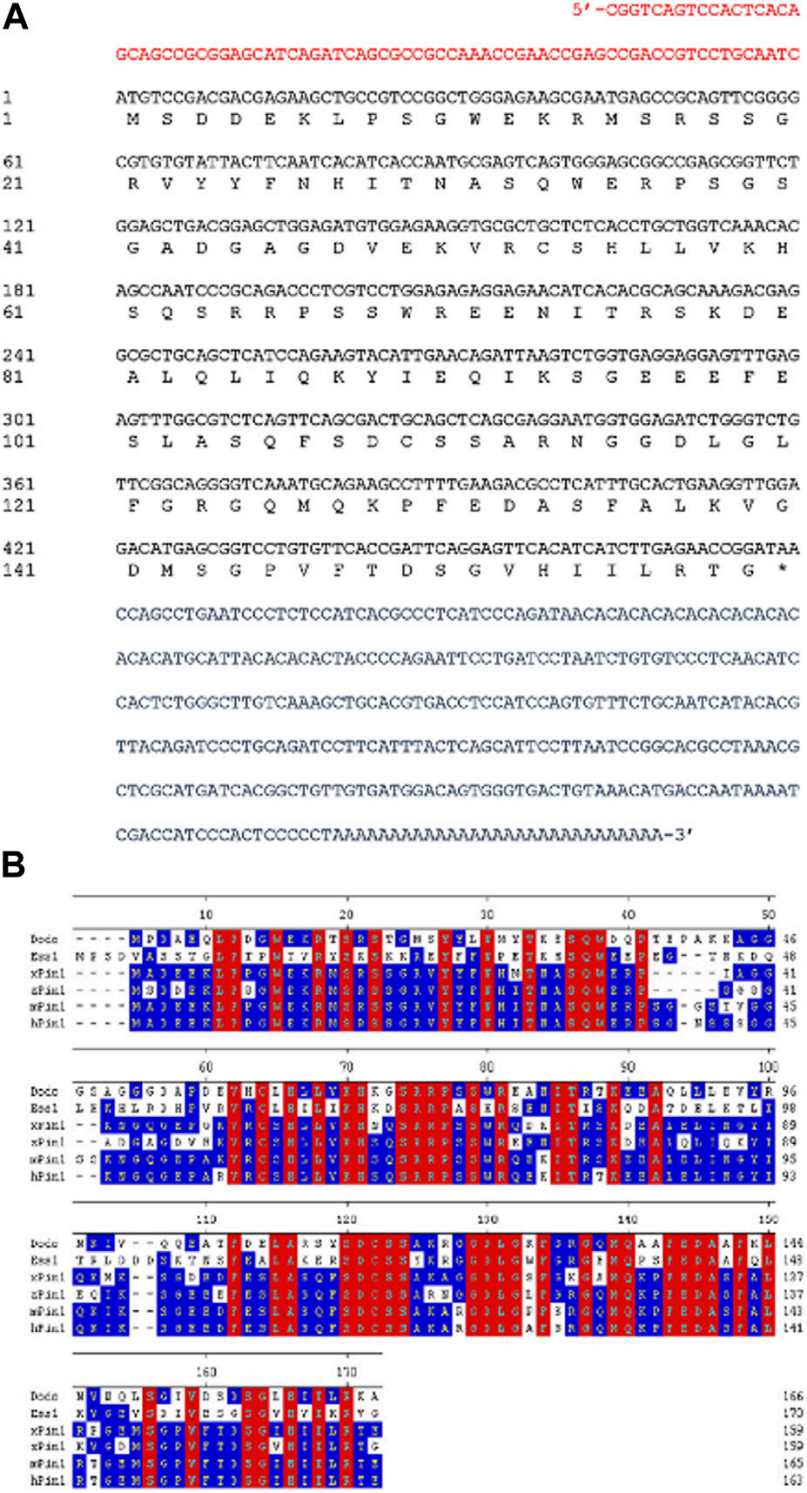
Sequence analysis of zebrafish Pin1. **(A)** The full-length cDNA sequence of zebrafish *pin1*. Red letters indicate 5′-UTR region; blue letters indicate 3′-UTR region; and black letters indicate the open reading frame. **(B)** Amino acid sequence alignment of Pin1 in *Drosophila* (dodo), budding yeast (Ess1), *Xenopus* (xPin1), zebrafish (zPin1), mouse (mPin1) and human (hPin1). Conserved residues in all species are highlighted in red. Residues with at least 50% identity across species are highlighted in blue. The numbers refer to amino acid positions of zebrafish Pin1.

To study *pin1* function in zebrafish, a full-length zebrafish *pin1* was cloned using Rapid Amplification of cDNA Ends (RACE) from total mRNA of adult zebrafish. The coding sequence of zebrafish *pin1* consists of a 480-bp open reading frame, an 81-bp 5′UTR and a 342-bp 3′UTR (Figure 1A). The cDNA of zebrafish *pin1* encodes a 159-amino acid protein that shares 79%, 79% and 78% identity with human, mouse and *Xenopus* Pin1, respectively (Figure 1B). Given the substantial similarity observed, it is probable that zebrafish Pin1, akin to other proteins within this family, will adopt a similar structural arrangement. This arrangement is expected to comprise a WW domain, serving as the binding/recognition domain, and a PPIase domain, functioning as the catalytic domain, which in turn will likely enable the isomerization of phosphorylated substrates.

**FIGURE 2:**
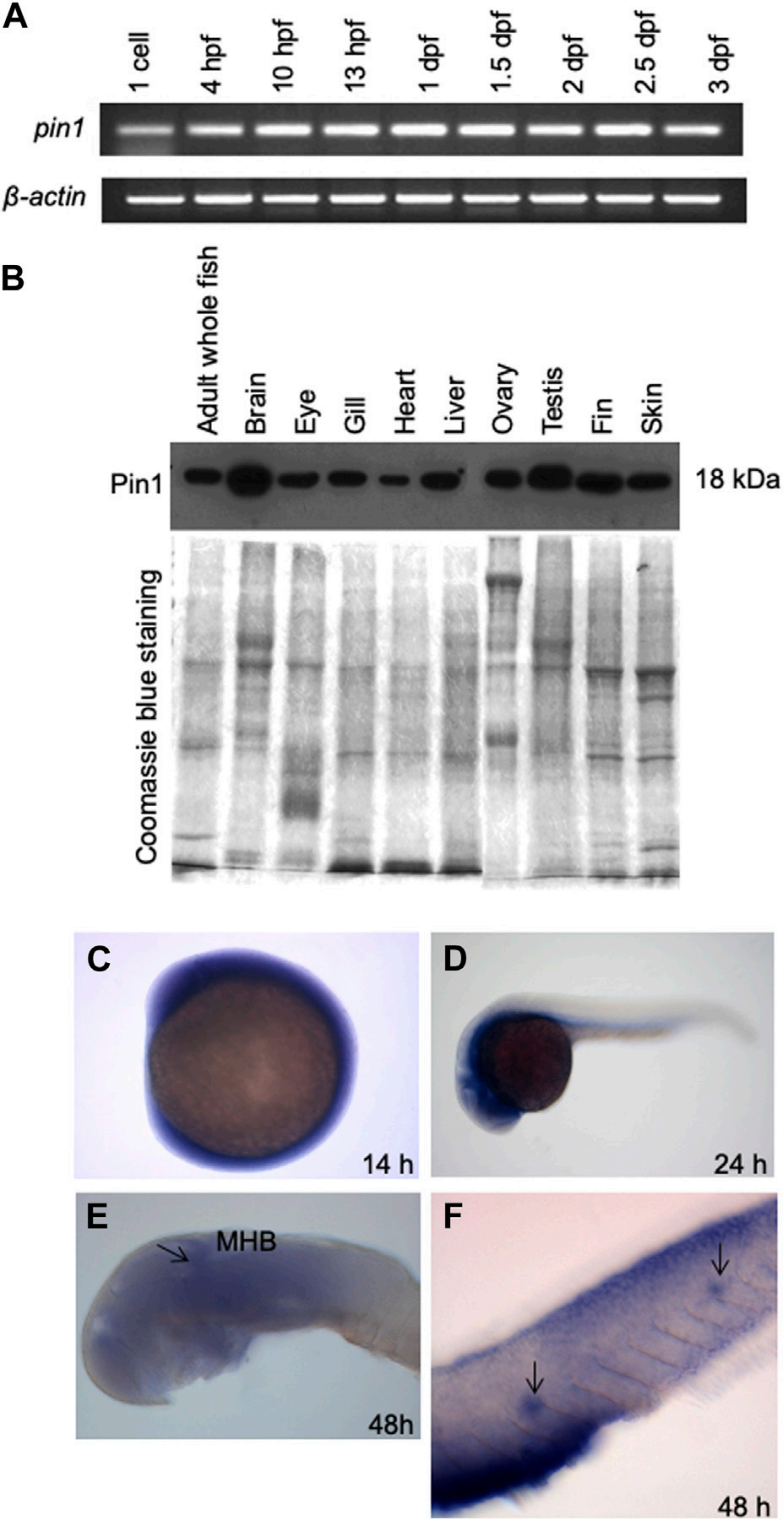
The spatial and temporal expression patterns of zebrafish Pin1. **(A)** Semiquantitative RT-PCR analysis of *pin1* expression in different stages of zebrafish development. *β-actin* served as an internal control. **(B)** Western blot analysis of Pin1 expression in adult zebrafish tissues. Coomassie Blue staining served as internal control. Each lane contains 30 µg of total protein. **(C–F)** WISH analysis of *pin1* expression in 14 hpf embryo **(C)**, 24 hpf **(D)** and 48 hpf embryo **(E,F)**. Arrow in **(E)** indicate midbrain-hindbrain boundary (MHB). Arrows in **(F)** mark the neuromasts staining in the PLL system.

Semiquantitative RT-PCR analysis of *pin1* in zebrafish embryos showed the presence of this transcript as early as 1-cell stage, suggesting that *pin1* is maternally deposited and remains relatively constant until 72 hpf (Figure 2A). Western blot analysis of Pin1 distribution in adult zebrafish tissues revealed that its ubiquity, with higher levels observed in the brain and testis (Figure 2B). In addition, whole-mount *in situ* hybridization (WISH) of *pin1* in zebrafish embryos showed that although the expression was ubiquitous early on, it became largely restricted to the developing brain by 24 hpf and 48 hpf (Figures 2C–E). These expression results are consistent with those in the previous report (Ibarra et al., 2017). The expression of *pin1* is also detected in the PLL neuromasts at 48 hpf (Figure 2F). Notably, sense *pin1* mRNA transcripts were not detected in the PLL neuromasts of 48 hpf embryos (Supplementary Figure S1). The specific expression of Pin1 in proliferating cells and neuromasts prompted us to investigate the role of Pin1 in neuronal development.

### Interference with Pin1 function in zebrafish embryos disrupts the formation of mechanosensory hair cells

**FIGURE 3:**
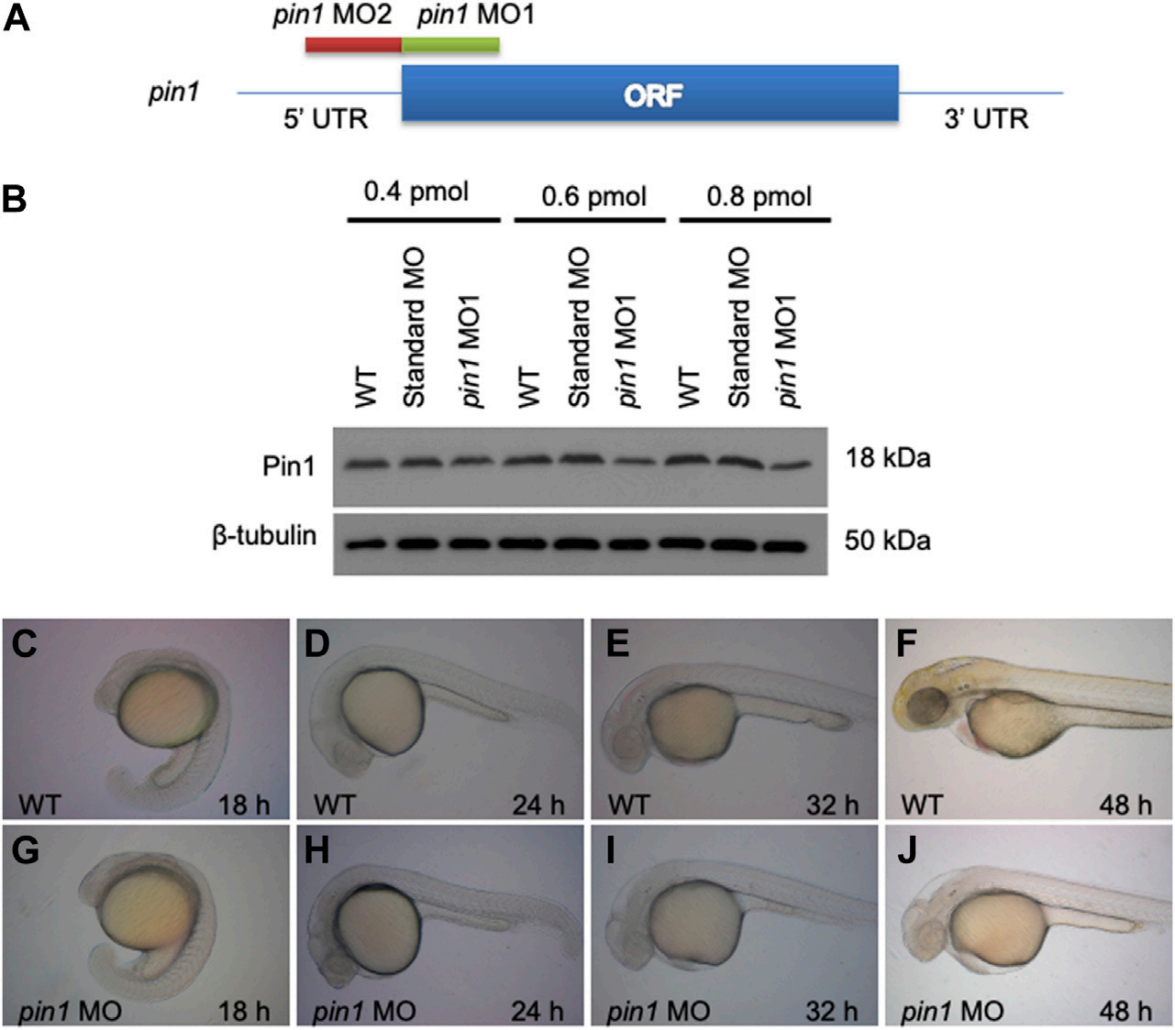
Zebrafish embryonic developmental delay in *pin1* morphants. **(A)** The design scheme of *pin1* MO1 and MO2. ORF: Open reading frame. **(B)** Pin1 protein levels in zebrafish embryos injected with standard control MO and *pin1* MO at the concentration indicated. β-tubulin was used as loading control. **(C–J)** Depletion of Pin1 results in embryonic developmental delay.

Two anti-sense morpholino oligonucleotides (MOs) were designed and used to target the translation initiation region (*pin1* MO1) and the 5′–UTR region (*pin1* MO2) of the *pin1* transcripts, aiming to knockdown Pin1 function in zebrafish embryos (Figure 3A). We firstly conducted an initial screen using both MO1 (Figure 3B) and MO2 (Supplementary Figure S2). Briefly, we observed that all zPin1 MO-injected embryos displayed developmental delay, and this delay was found to be dose-dependent. Increasing the injected morpholino amount from approximately 0.3 pmol–0.6 and even 0.8 pmol resulted in more evident phenotypes of developmental delay. Although both *pin1* MOs, but not the standard control MO, reduced the level of Pin1 in zebrafish embryos (Figure 3B; Supplementary Figure S2). However, when the injected morpholino amount reached 0.8 pmol, all embryos exhibited toxic effects with global apoptosis, malformed brain, and edema. As a result, we had to limit the amount of morpholino to a maximum of 0.6 pmol. On the other hand, MO2 did not exhibit as pronounced of a knockdown effect at 0.6 pmol; therefore, we primarily used MO1 to study the phenotypes. MO2 was mainly utilized as a morpholino specificity control.

Depletion of Pin1 in zebrafish resulted in severe developmental delay of up to 2 h in 24 hpf embryos and 6 h in 48 hpf embryos (Figures 3C–J). Since p53 is a known substrate of Pin1, the developmental delay observed in morphant embryos could be attributed to the role of Pin1 in cell cycle regulation and apoptosis (Mantovani et al., 2004; Becker and Bonni, 2007; Ryo et al., 2007). In addition, p53 is also involved in some off-target effects of MO. Concurrent knockdown of p53 specifically attenuates the off-targeting cell death induced by MO while p53 MO does not affect specific loss of gene function (Robu et al., 2005). To eliminate the potential secondary effects of p53, subsequent analyses using *pin1* MOs were performed in the presence of *p53* MO (Eisen and Smith, 2008).

**FIGURE 4:**
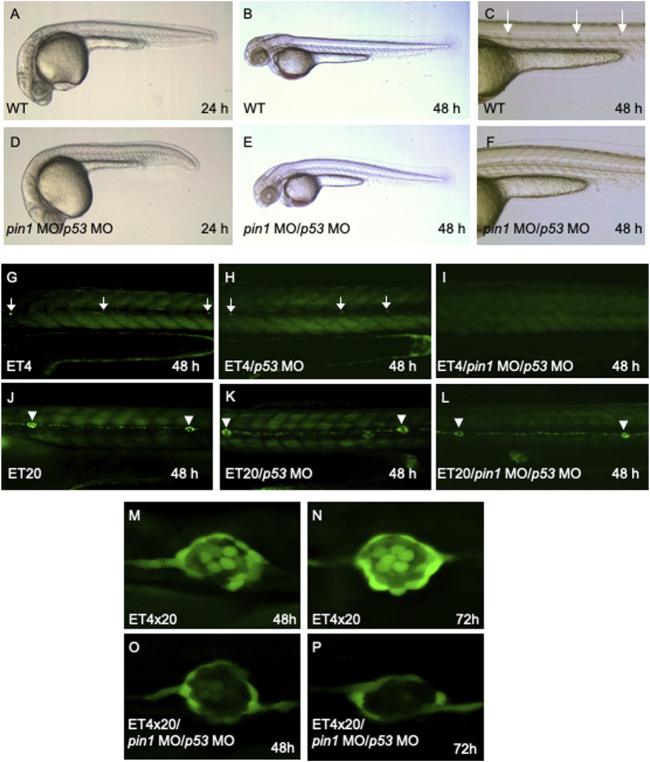
Specific PLL neuromasts hair cells defects in *pin1* morphants. **(A–F)** Co-injection of *pin1* MO with *p53* MO results in slight developmental delay and defective neuromasts hair cells formation in zebrafish embryos. Arrows in **(C)** indicate posterior neuromasts. **(G–I)** Analysis of neuromasts cells in *pin1* MO/*p53* MO injected ET4 embryos. Arrows in **(G, H)** indicate neuromasts hair cells along the PLL. **(J–L)** Analysis of mantle cells in *pin1* MO/*p53* MO injected ET20 embryos. Arrowheads in **(J–L)** indicate neuromasts mantle cells. **(M–P)** Analysis of hair cells and mantle cells in 48 hpf and 72 hpf double transgenic (ET4x20) embryos.

Co-injection of *pin1* MO/*p53* MO resulted in developmental delay of 2 h in 24 hpf embryos and reduced the developmental delay to 4 h in 48 hpf embryos (Figures 4A, B, D, E) without any hint of off-target effects associated with MOs. Importantly, morphants displayed a reduced number of posterior neuromasts (Figures 4C, F), suggesting a role of Pin1 in neuromasts formation. We next proceeded to knock down *pin1* in two *Tol2* enhancer trap lines, ET4 and ET20, with GFP expression in the hair cells and mantle cells, respectively (Parinov et al., 2004). The *pin1 MO/p53 MO* injected ET4 fish displayed a marked decrease in the number of anterior neuromast hair cells, and a complete loss of these cells along the PLL at 48 hpf (Figure 4I). However, ET20 injected with *pin1* MO/*p53* MO displayed the full complement of neuromasts mantle cells (Figure 4L). In both ET lines, *p53* MO had no observable effects in neuromasts development (Figures 4H, K).

The specific loss of hair cells, but not mantle cells by 72 hpf was clearly demonstrated when *pin1* MO/*p53* MO were injected into double transgenic embryos derived from crossing ET4 and ET20 (ET4x20, Figures 4M–P). Furthermore, WISH for *atoh1a* transcripts at 36 and 48 hpf showed that both *atoh1a* positive proneuromasts and deposited neuromasts were present in Pin1 morphants (Supplementary Figure S3), suggesting that initial specification of neuromasts was not affected by the attenuation of Pin1 function. Taken together, the data suggest that Pin1 is required for the specification of hair cells, but not the mantle cells. Similar to *pin1*, loss of function of Nrd specifically resulted in the loss of hair cells in zebrafish (Sarrazin et al., 2006). Taken together, our data suggest a possible link between Pin1 and Nrd activity in regulating PLL hair cells formation in zebrafish.

To evaluate whether neuron determination process was affected, we utilized glial fibrillary acidic protein (GFAP) antibody to stain embryos. GFAP is an intermediate filament primarily expressed in astrocytes and radial glial cells of the central nervous system (CNS). Radial glial cells are neural stem cells from which most of neurons in brain are derived, either directly or indirectly. Supplementary Figure S4A and B showed an indicatical staining pattern, indicating that early neuron specification or determination remained unaffected. However, we suspected that Pin1 might interfere with neuron differentiation process. To further characterize zPin1 knockdown neuronal phenotypes, we performed *in situ* hybridization analysis using several markers, including neurogenin1 (ngn1), neuroM, her4, neuroD. The choice of neuroD for analysis was due to the observed interaction between zPin1 and NeuroD, as well as the key role of neuroD as a neuron differentiation factor. The *in situ* hybridization results, presented in Supplementary Figure S4, showed a defective neuroD expression pattern, particularly with the loss of neuroD transcripts, but not other markers, in the midbrain and hindbrain region. These findings suggest that zPin1 may impact neuroD expression, thereby influencing the process of neuron differentiation.

### The zebrafish Pin1 interacts with Nrd and affect Nrd protein stability

**FIGURE 5:**
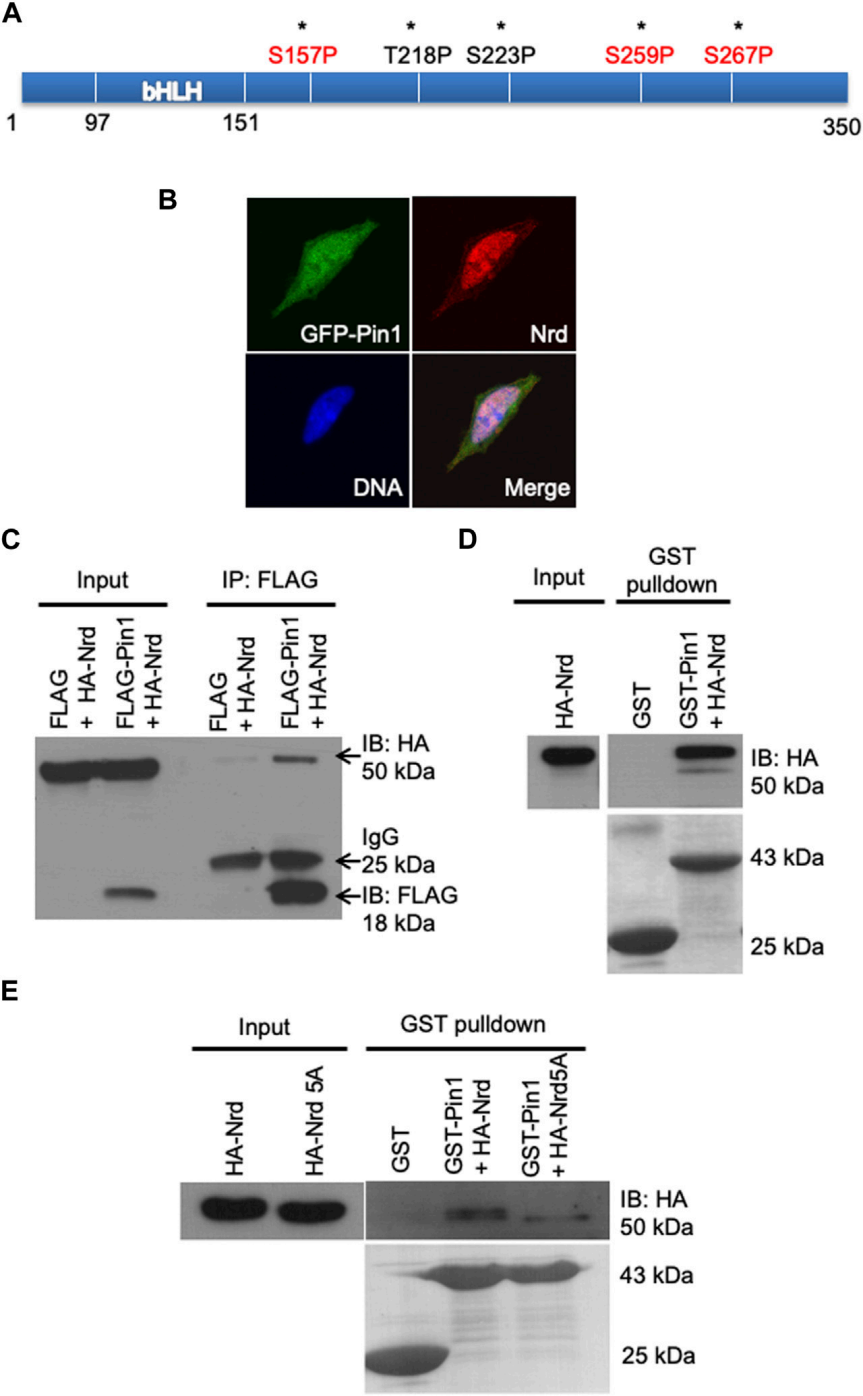
Zebrafish Pin1 interacts with Nrd via pSer/Thr-Pro motifs. **(A)** A schematic illustration of the potential Pin1-binding sites in Nrd. **(B)** Zebrafish Pin1 and Nrd co-localizes in the SH-SY5Y cell nucleus. DNA (blue), zebrafish Pin1 (green), Nrd (red). **(C,D)** Zebrafish Pin1 interacts with Nrd *in vivo* and *in vitro.* **(E)** The interaction of HA-Nrd5A mutant and zebrafish Pin1 is compromised.

Since Pin1 specifically regulates the phosphorylated proteins through their pSer/pThr-Pro motifs, to investigate the potential Pin1 binding sites of Nrd, we performed sequence analysis of Nrd and identified five potential Pin1-binding Ser/Thr-Pro motifs (asterisks, Figure 5A). Among these motifs, the consensus Ser157, Ser259, and Ser267 residues (highlighted in red, Figure 5A) were known to be phosphorylated by ERK2 or GSK3β in *Xenopus* (Marcus et al., 1998; Moore et al., 2002; Khoo et al., 2003). This led us to hypothesize that Pin1 could interact physically with Nrd to regulate its function during differentiation of mechanoreceptors. To test this hypothesis, we co-transfected GFP-tagged zebrafish Pin1 and HA-tagged Nrd were transiently co-transfected into neuroblastoma SH-SY5Y cells. The results, as shown in Figure 5B, revealed that NeuroD was predominantly localized in nucleus (Figure 5B, labelled in red). As for Pin1 (Figure 5B, labelled in green), the images showed mostly of GFP-Pin1 resided in nucleus, with some signal being detected in cytoplasm. The merged image revealed that these two proteins co-localized in the nucleus, suggesting that they had the required proximity to interact.

Subsequently, we conducted an *in vivo* co-immunoprecipitation (Co-IP) FLAG pulldown experiment was performed. HEK 293T cells were co-transfected with FLAG-zPin1 and HA-NeuroD, and then we performed the FLAG pulldown assay using harvested whole cell lysates. We utilized anti-FLAG and anti-HA antibodies to detect zPin1 and NeuroD, respectively. As depicted in Figure 5C (arrow), our results demonstrated that FLAG-zPin1 successfully pulled down HA-NeuroD, indicating that an *in vivo* interaction of between zPin1 and NeuroD. This suggests that zPin1 may play a role in post-translational regulation of NeuroD and forming the basis for all our subsequent studies. Next, we utilized recombinant glutathione S-transferase (GST)-zebrafish Pin1 to further demonstrate the interaction. The results showed that GST-Pin1 could bind to HA-tagged Nrd overexpressed in HEK293T cells, providing evidence of an *in vitro* interaction between zebrafish Pin1 and Nrd (Figures 5C, D).

To further investigate the phosphorylation-dependent interaction between Pin1and Nrd via Ser/Thr-Pro motifs, we next conducted a screening using site-directed mutagenesis. Substituting all five Ser-Thr/Pro motifs in Nrd with alanine (HA-Nrd5A) significantly attenuated zebrafish Pin1 binding, indicating a motif specific interaction between zebrafish Pin1 and Nrd (Figure 5E). Notably, substantial binding between Pin1 and Nrd persisted even when up to four of the Ser/Thr-Pro motifs in Nrd were substituted with alanine (Supplementary Figure S5). In addition, the interaction between zebrafish Pin1 and HA-Nrd was reduced in HEK 293T cell lysates treated with calf intestine alkaline phosphatase (CIAP) (Supplementary Figure S6). Taken together, these results collectively demonstrate that zebrafish Pin1 interacts with Nrd via all five Ser/Thr-Pro motifs in Nrd and that this interaction is phosphorylation dependent.

**FIGURE 6:**
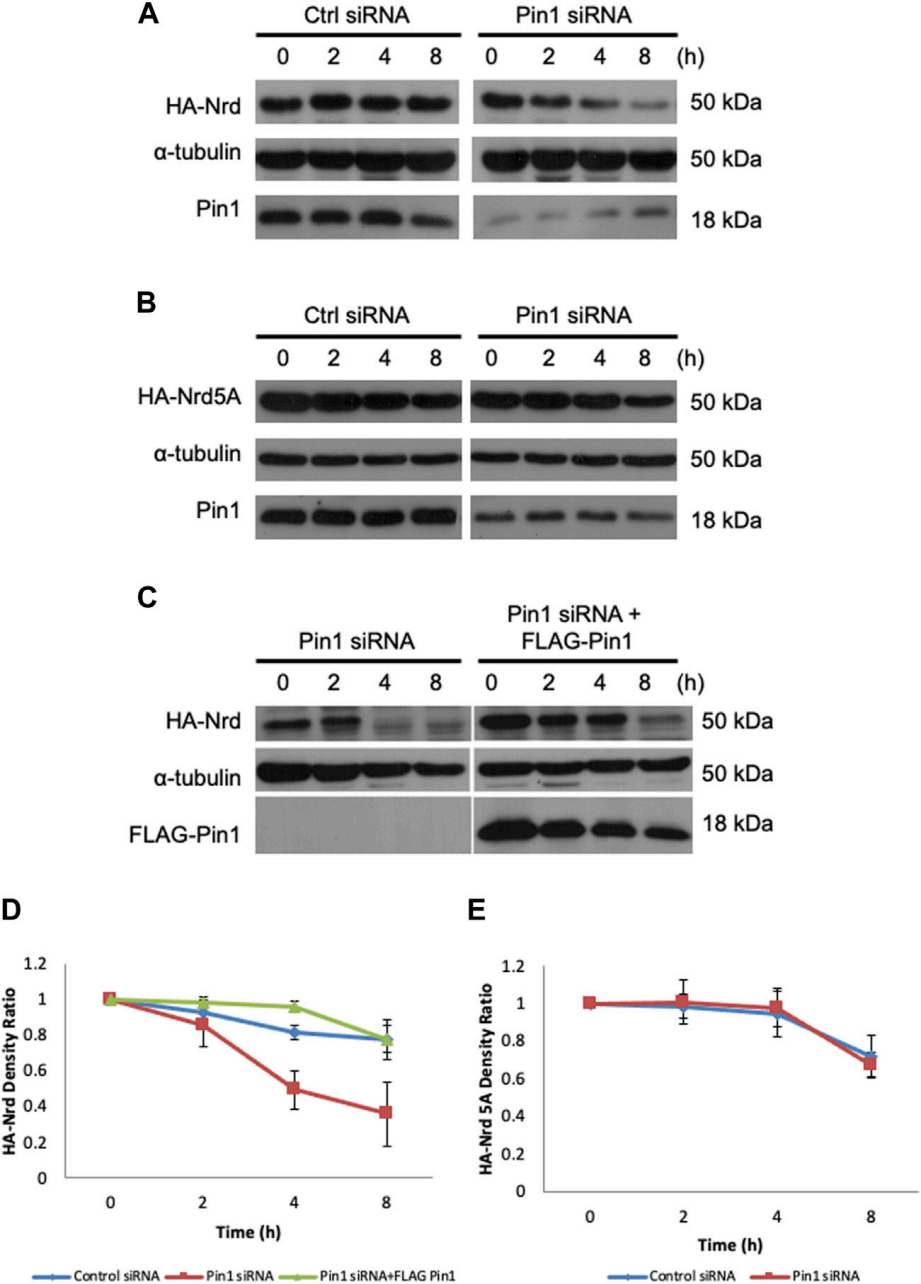
Pin1 regulates Nrd stability. Protein stability assay of HA-Nrd in HEK 293T cells stably expressing control siRNA and Pin1 siRNA. **(A)** HA-Nrd degraded at a higher rate in Pin1 knock-down background in HEK 293T cell line. **(B)** HA-Nrd5A levels remained stable in Pin1 knock-down background in HEK 293T cells. **(C)** Overexpression of FLAG-tagged zebrafish Pin1 enhanced Nrd stability. α-tubulin was used as an internal control. **(D)** Quantification of HA-Nrd levels in **(A,C)** normalized to α-tubulin levels. **(E)** Quantification of HA-Nrd levels in **(B)** normalized to α-tubulin levels. Error bars represent ±SEM.

The interaction between Pin1 and its substrates has been demonstrated to be crucial in regulating substrates stability and turnover (Lu and Zhou, 2007). To explore the impact of Pijn1 on Nrd, we next examined the stability of HA-Nrd in HEK 293T cells with stably expressing human Pin1 siRNA and control siRNA, treated with cycloheximide. HA-Nrd in Pin1-depleted HEK 293T cells showed reduced stability with a higher turnover rate compared to control cells (Figure 6A). Similar results were observed in Pin1 knockout MEF cells (Supplementary Figure S7A). Additionally, the stability of HA-Nrd5A, which has compromised for binding with zebrafish Pin1, was protected from degradation in the absence of Pin1 (Figures 6B, E). We observed slight variations in the levels of Pin1 (under siRNA treatments) between Figures 6A, B, which can be attributed to differences in the exposure time of the films during experimentation. However, it is important to note that these variations fall within an acceptable range of variation. Overall, our results and conclusions remain consistent. Importantly, the stability of HA-Nrd was rescued by re-introduction of FLAG-tagged zebrafish Pin1 (Figures 6C, D) and adenovirus encoding human Pin1 (Supplementary Figure S7B) in HEK 293T Pin1 siRNA cells. These findings indicate that Pin1 plays a direct and critical role in modulating Nrd stability. Taken together, our data suggest that Pin1 functions in Nrd dependent neuronal specification events by regulating Nrd stability.

## Discussion

Studies of the biological role of Pin1 in model organisms revealed that Pin1 is important in regulating cell growth in *T. brucei*, flowering mechanism in *Arabidopsis* and is indispensable in regulating cell proliferation and neurodegeneration in mice (Liou et al., 2002; Liou et al., 2003; Goh et al., 2010; Wang et al., 2010) Pin1 knockout mice displayed age-dependent neurodegeneration, which is linked to its role in regulating tau protein (Liou et al., 2003). However, it remains unclear if Pin1 plays a role in neuronal specification during early development in vertebrates.

The high homology between zebrafish and mammalian Pin1 (Figure 1B) and the presence of *pin1* expression in the zebrafish PLL neuromasts (Figure 2F) provided us with an opportunity to investigate the *in vivo* role of Pin1 in neuronal specification during early vertebrate development. The analysis of the spatial and temporal distribution of Pin1 in adult zebrafish through Western blot analysis (Figures 2A, B) is consistent with its known roles of Pin1 in neurodegeneration, primordial germ cell proliferation and spermatogonial depletion in mice (Liou et al., 2002; Atchison et al., 2003; Atchison and Means, 2003).

Both translational blocking MOs reduced Pin1 levels in zebrafish embryos effectively (Figure 3B; Supplementary Figure S2). Whereas traces of *pin1* expression remained in morphants, the loss of hair cells is strikingly consistent (Figure 4). Concurrent knockdown of Pin1 and p53 in zebrafish resulted in reduced developmental delay compared to Pin1 knockdown alone (Figures 4A–F). Importantly, co-injection of *pin1* MO*/p53* MO into *Tol2* transposon-mediated enhancer trap lines, ET4 and ET20, with GFP expression in the hair cells and mantle cells respectively (Parinov et al., 2004), confirmed that Pin1 is required for the development of mechanosensory hair cells but not mantle cells in zebrafish embryos (Figures 4G–P). Interestingly, this phenotype resembles the one described previously in Nrd morphants (Sarrazin et al., 2006).

Due to the pleiotropic function of Pin1, overexpression of *pin1* mRNA alone resulted in more severe developmental defect than *pin1* MOs. Co-injection of *pin1* mRNA in different dosage with *pin1* MO into 1-cell stage embryos did not lead to rescue of lateral line neuromasts, but rather presented itself with a complicated phenotype. Furthermore, in our attempt to perform rescue in a tissue restricted manner, control cells were transplanted from a donor embryo to a host during gastrula stage (6 hpf) with a tracer positioned to be incorporated into the lateral line at 48 hpf. However, in the presence of *pin1* MO and/or *pin1* mRNA, these cells could no longer be targeted to the lateral line. Despite this, our results indicate that *pin1* MOs targeting different regions of the zebrafish *pin1* transcripts produced similar phenotypes in the lateral line neuromasts.

Regarding to the function of zebrafish Pin1 in mechanosensory cells specification, we have identified Nrd as a novel Pin1 substrate. The interaction between zebrafish Pin1 interacts and Nrd occurs through all five Ser/Thr-Pro motifs in a phosphorylation-dependent manner (Figure 5). All NeuroD single and double mutants interacted with zPin1 and displayed a similar zPin1 binding ability to wild-type NeuroD. The 3A mutant of NeuroD, with other phosphorylation sites investigated, showed a minor reduction in interaction ability. The 4A mutants, generated by mutating two additional Ser/Thr sites (Thr218, Ser223), also displayed a comparable decrease in zPin1 binding (data not shown). In contrast, the 5A NeuroD mutant, with Ser-to-Ala substitutions on all five Ser/Thr-Pro motifs, exhibited a significantly reduced binding to zPin1 (Figure 5). These findings indicate that the interaction between NeuroD and zPin1 relies on the synergistic effect of multiple Ser/Thr-Pro motifs rather than any specific one.

Although some residual interaction between Pin1 and Nrd was detected even after replacement of all five Ser/Thr-Pro motifs in Nrd with Ala, this observation is not surprising as other Pin1 substrates including p53 and p73 maintained trace binding to Pin1 even when all of their Ser/Thr-Pro sites were mutated (Zheng et al., 2002; Oberst et al., 2005). In this study, our data highlight the importance of all five Ser/Thr-Pro motifs of Nrd for interaction with Pin1. However, further studies are required to address the question of how all five Ser/Thr-Pro motifs in Nrd synergize to regulate the interaction with Pin1. Due to the dynamic nature of phosphorylation processes *in vivo*, it is challenging to pinpoint the specific site(s) of NeuroD phosphorylation during developmental stages. Additional studies will be necessary to address this aspect and further elucidate the intricacies of NeuroD phosphorylation.

Previous studies have demonstrated that the *cis-trans* isomerization activity of Pin1 on its substrates is important in regulating substrate stability (Lu and Zhou, 2007). In our study, we observed that the interaction of Pin1 with Nrd on Ser/Thr-Pro motifs stabilizes Nrd (Figure 6). Given the high sequence similarity between the mammalian Pin1 and zebrafish Pin1, both proteins may act interchangeably in regulating Nrd stability. Our stability assay showed that Pin1 can stabilize NeuroD because in both Pin1 knockdown 293T cells and Pin1 knockout MEFs cells, overexpressed NeuroD had a shorter half-life compared to that in wild type cells. Pin1 functions as a molecular switch, enhancing the stability of its substrates or preventing protein degradation of its substrates via ubiquitin-mediated degradation pathway (Lu and Zhou, 2007). The protein turnover rate of two other bHLH factors, MyoD and Ngn1, has been shown to be regulated by the ubiquitin-mediated degradation pathway (Song et al., 1998). Therefore, we speculate that the interaction of Pin1 and Nrd protects Nrd from degradation via ubiquitin-mediated degradation pathway.

NeuroD phosphorylation has been demonstrated in neuronal cells and pancreatic beta cells (Khoo et al., 2003; Dufton et al., 2005), impacting its transcriptional activity during insulin gene activation and neurogenesis (Marcus et al., 1998; Moore et al., 2002; Dufton et al., 2005). Both GSK3β and ERK have been shown to phosphorylate NeuroD. GSK3β phosphorylates Ser274, inhibiting NeuroD activity and negatively regulating neuronal differentiation (Marcus et al., 1998). In contrast, ERK2 phosphorylates Ser162, Ser259, Ser266, and Ser274, but its effects may be context-dependent. Mutations in S266A and S274A inhibit the ability of mouse NeuroD to activate the insulin gene, while the same mutations in *Xenopus* NeuroD increase the ability to form ectopic neurons (Khoo et al., 2003; Dufton et al., 2005). The integrity of the GSK3β consensus phosphorylation site is crucial for regulating NeuroD phosphorylation (Moore et al., 2002). The presence of the GSK3β consensus phosphorylation motif in zebrafish NeuroD suggests GSK3β may also regulate phosphorylated NeuroD activity in zebrafish.

In summary, this study reveals the novel role of Pin1 in neuromasts specification during vertebrate development, using zebrafish as a model organism. We also identified Nrd as a new substrate of zebrafish Pin1, and our finding demonstrate that the interaction between zebrafish Pin1 and Nrd via the Ser/Thr-Pro motifs enhances Nrd stability. These results highlight the role of Pin1 in regulating proneural bHLH factors in neuronal specification during early vertebrate development, in addition to its known role in regulating age-dependent neurodegeneration in mice.

## Data Availability

The original contributions presented in the study are included in the article/Supplementary Material; further inquiries can be directed to the corresponding authors.

## Abbreviations

ERK2, extracellular-signal-regulated kinase 2; GSK3β, glycogen synthase-3; RT-PCR, reverse transcription-polymerase chain reaction; UTR, untranslated region; GFP, green fluorescent protein; HA, hemagglutinin; HEK, human embryonic kidney; MEF, mouse embryonic fibroblast; siRNA, small interfering RNA; FBS, fetal bovine serum; BSA, bovine serum albumin; PBS, phosphate buffer saline; SDS-PAGE, sodium dodecyl sulfate polyacrylamide gel electrophoresis; WISH, whole-mount *in situ* hybridization; PLL, posterior lateral line; MO, morpholino oligonucleotides; hpf, hours post-fertilization; ORF, open reading frame.
