# Supplementary material for: The prolyl isomerase Pin1 stabilizes NeuroD during differentiation of mechanoreceptors
Source: Front Cell Dev Biol. 2023 Sep 18;11:1225128. doi: 10.3389/fcell.2023.1225128 (PMC10543749; doi:10.3389/fcell.2023.1225128)
Supplement: Supplementary file 1 [file DataSheet2.PDF]

Supplementary Figure Legends

Figure S1. WISH analysis using sense *pin1* RNA probe. WISH analysis of *pin1* expression in 48 hpf embryo using sense *pin1* RNA probe. (A) Lateral view, (B) Dorsal view.

Figure S2. *pin1* MO2 suppresses Pin1 protein translation in zebrafish embryos. Pin1 protein levels in zebrafish embryos injected with *pin1* MO2 at the concentration indicated. β-tubulin was used as loading control.

Figure S3. WISH for *atoh1a* transcripts at 36 hpf and 48 hpf. (A, B) Wildtype embryos, (C, D) *pin1* MO/*p53* MO injected embyros. i) dorsal view of 4th ventricle; ii) lateral view of lateral line where proneuromast (white arrows) and deposited neuromasts (black arrows) are highlighted.

Figure S4. Figure 3.13 Expression of marker genes in zPin1 morphant embryos. Wild type embryos and zPin1 morphant were collected at 48 h and analyzed for expression of different makers. GFAP staining (A, B), ngn1 (C, D), neuroM (E, F), her4 (G, H), neuroD (I, J), at 100 x magnification, neuroD at 200 x (K, L),

Figure S5. Interaction of zebrafish Pin1 with HA-Nrd mutants. (A, B) Single and double mutations of Ser to Ala in Nrd have no effects on the interaction between zebrafish Pin1 and Nrd. (C) Nrd3A and 4A mutants display slight reduction of interaction with zebrafish Pin1.

Figure S6. The interaction between zebrafish Pin1 and Nrd is phosphorylation dependent. Calf intestine alkaline phosphatase (CIAP) treated HEK 293T whole cell lysates transfected with HA-Nrd were subjected to GST-pulldown assay and analysed by immunublotting with anti-HA antibodies.

Figure S7. Pin1 regulates Nrd stability. (A) Nrd degrades at a higher rate in Pin1 knock-out background in MEFs cell line. (B) Overexpression adenovirus encoding Pin1 enhanced Nrd stability.
