# Supplementary figures and images for: The prolyl isomerase Pin1 stabilizes NeuroD during differentiation of mechanoreceptors
Source: Front Cell Dev Biol. 2023 Sep 18;11:1225128. doi: 10.3389/fcell.2023.1225128 (PMC10543749; doi:10.3389/fcell.2023.1225128)

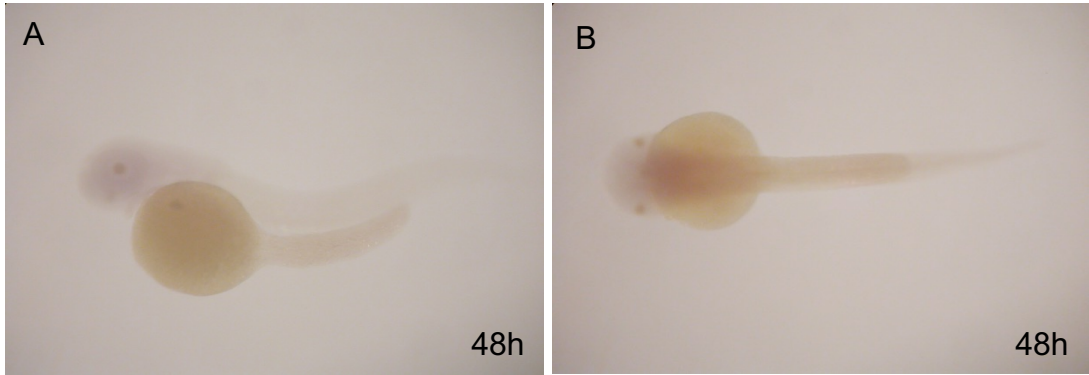

**Figure S1**

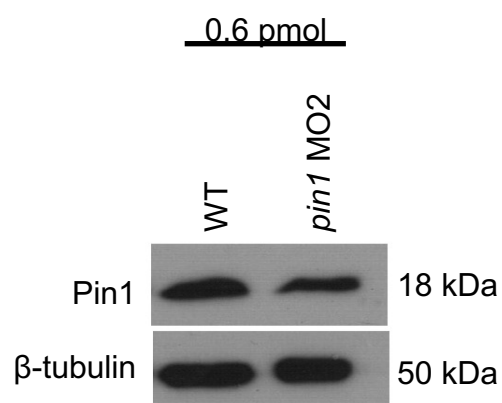

**Figure S2**

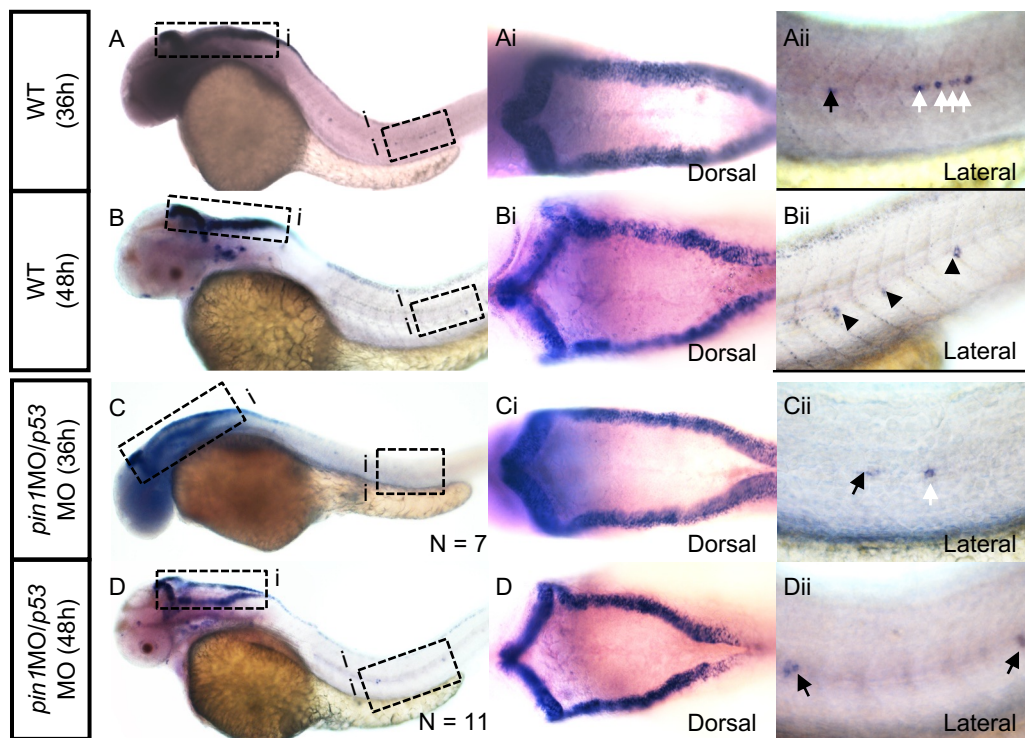

**Figure S3**

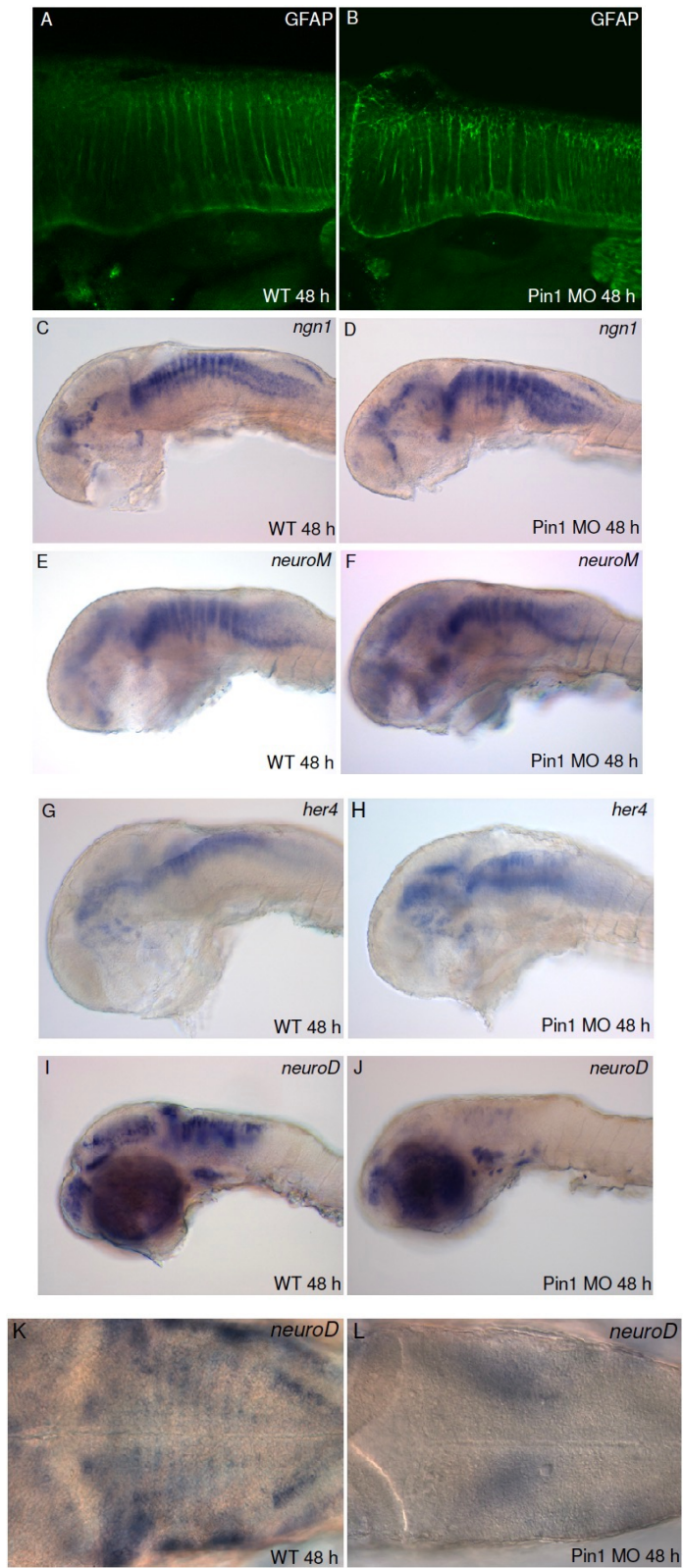

**Figure S4**

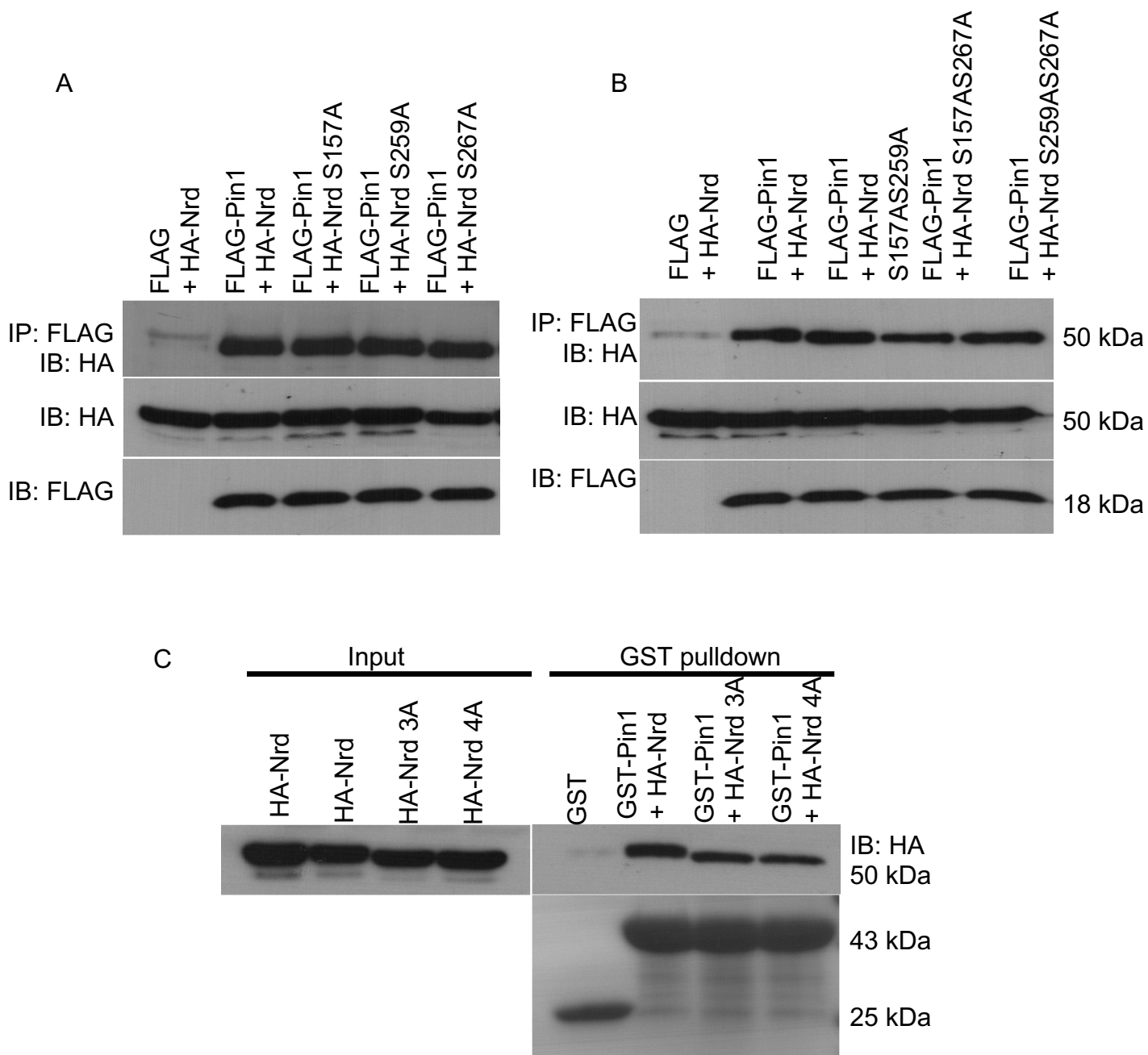

**Figure S5**

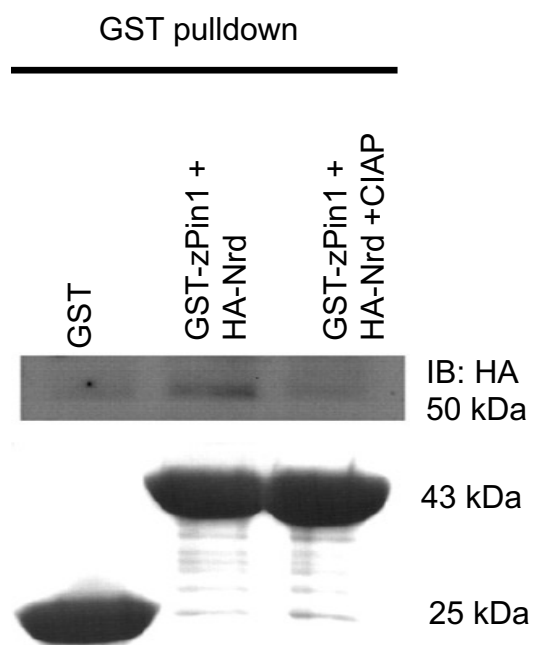

**Figure S6**

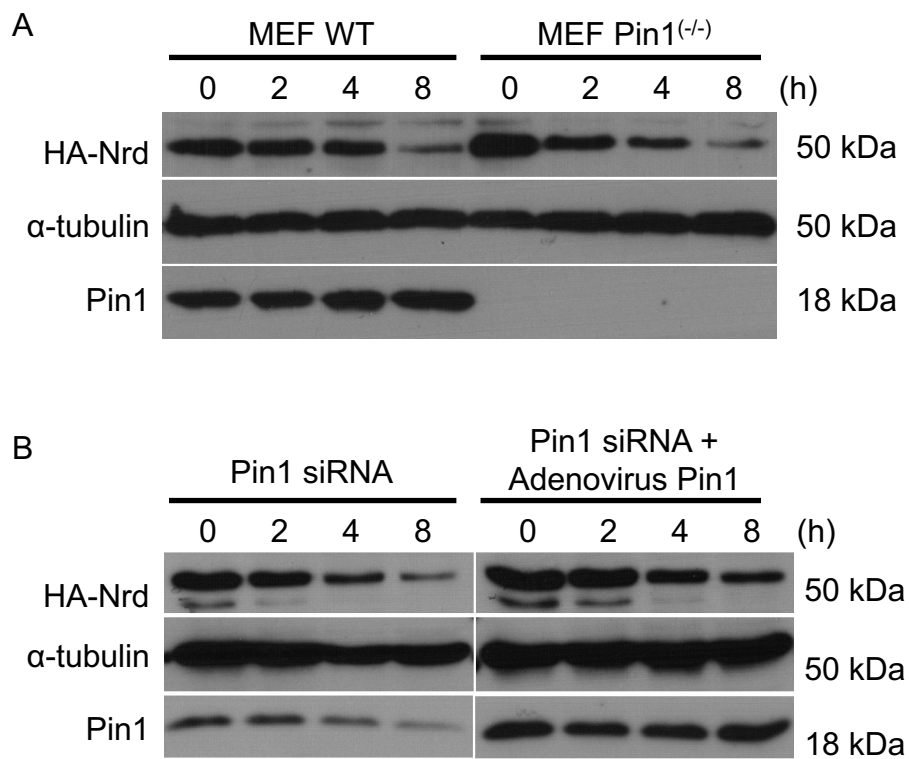

**Figure S7**

Supplement: Supplementary file 2 [file DataSheet1.PDF]
